# Optimization of Selective Assembly for Shafts and Holes Based on Relative Entropy and Dynamic Programming

**DOI:** 10.3390/e22111211

**Published:** 2020-10-26

**Authors:** Mingyi Xing, Qiushuang Zhang, Xin Jin, Zhijing Zhang

**Affiliations:** 1 College of Electromechanical Engineering, Qingdao University of Science and Technology, Qingdao 266061, China; myxing1212@126.com; 2 School of Instrumentation and Optoelectronic Engineering, Beihang University, Beijing 100083, China; 3 School of Mechanical Engineering, Beijing Institute of Technology, Beijing 100081, China; jinxinbit@163.com (X.J.); zhzhj@bit.edu.cn (Z.Z.)

**Keywords:** selective assembly, optimization, precision instrument, relative entropy, dynamic programming

## Abstract

Selective assembly is the method of obtaining high precision assemblies from relatively low precision components. For precision instruments, the geometric error on mating surface is an important factor affecting assembly accuracy. Different from the traditional selective assembly method, this paper proposes an optimization method of selective assembly for shafts and holes based on relative entropy and dynamic programming. In this method, relative entropy is applied to evaluate the clearance uniformity between shafts and holes, and dynamic programming is used to optimize selective assembly of batches of shafts and holes. In this paper, the case studied has 8 shafts and 20 holes, which need to be assembled into 8 products. The results show that optimal combinations are selected, which provide new insights into selective assembly optimization and lay the foundation for selective assembly of multi-batch precision parts.

## 1. Introduction

The demand for precision instruments in various industries is increasing. The assembly accuracy of precision instruments is strict, which is at the level of a micron, or even a nanometer. At present, the assembly accuracy of precision instruments is guaranteed by two aspects: controlling the machining accuracy of parts through tolerance analysis and adjusting the assembly process as per monitoring data. Due to the limitations of machining ability and cost, it is impossible to guarantee assembly accuracy by unboundedly improving machining accuracy. Thus, adjustment of the assembly process is an effective method to ensure the assembly accuracy of precision machinery.

**Figure 1:**
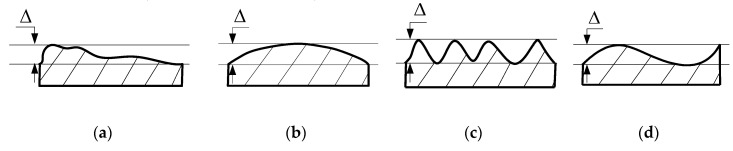
Four types of surface features on surface *P*_1_; (**a**) case a; (**b**) case b; (**c**) case c; (**d**) case d.

**Figure 2:**
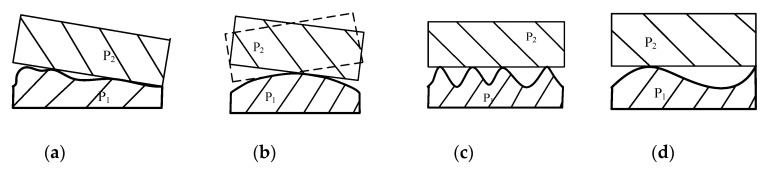
Assembly errors caused by part *P*_1_; (**a**) case a; (**b**) case b; (**c**) case c; (**d**) case d.

Adjustment of the assembly process includes adjustment position, repair method, and selective assembly. In detail, the adjustment position method is used to adjust the relative position between parts, such as assembly angle; the repair method is to ensure the assembly accuracy by secondary processing, such as manual grinding, scraping, and filing; the selective assembly refers to the selection of appropriate parts in the key assembly process [1]. Among them, the selective assembly has good effect and low cost, and is widely used in actual assembly. Selective assembly is the method of obtaining high precision assemblies from relatively low precision components. The traditional selective assembly mainly includes the direct matching method, the grouping selection method, and the composite matching method. For precision instruments, the geometric error on mating surface is an important factor affecting assembly accuracy [2]. According to ASME Y14.5-2009 [3], geometric error is the deviation of dimension, shape, direction, position, and runout relative to ideal geometric parameters. Assembly errors are caused by form deviations, profile, orientation, waviness, and roughness. As Figure 1 shows, four parts with identical flatness produce completely different assembly effects, due to their different geometric shapes and spatial distribution. Figure 2 illustrates the assembly of an ideal part using these four surface types, and it can be seen that when P_2_ mates with P_1_, the assembly errors for four cases give different results.

For precision mechanical systems, geometric errors on mating surfaces directly affect the position of the part during assembly and change the contact state between the parts [4]. If the distribution of the geometric error is uniform, the assembly error is small and the contact state will be stable—as shown in case (c) in Figure 2. Furthermore, for shafts and holes of precision mechanical systems, the size and uniformity of assembly clearance will affect the assembly accuracy and stability. Therefore, the geometric error on mating surface must be considered in the selective assembly process, instead of simply using traditional selective assembly methods. Considering the characteristics of precision shafts and holes, such as small size, high rigidity, high machining precision, and good surface quality, this paper focuses on the influence of shape deviation on assembly.

## 2. Related Works

At present, researchers have mainly achieved optimization of selective assembly from two aspects: evaluation index and optimization algorithm. The appropriate evaluation index is the premise to determining the optimization objective.

There has been some work done on evaluation index, such as Taguchi’s mass loss function, square loss function, and process capability index, among others. Using Taguchi’s mass loss function as evaluation index, Kannan et al. searched the optimal grouping of selective assembly [5]. On this basis, the quality loss function of Taguchi theory is used as a characterization tool to build a customized product matching model by Tu Zhenyin [6]. Matsuura et al. extended the square loss function to the convex loss function and established the optimal grouping [7]. Based on process capability index, Lin et al. constructed an optimization model of matching parts assembly [8]. To solve the remanufacturing optimization matching problem, an assembly success rate and a remanufacturing resource utilization rate were presented as the assembly quality evaluation indexes [9]. Sun et al. considered the effect of closed loop on the fitted position and put forward three methods to improve selective assembly [10]. Asha et al. proposed a grouping selection method suitable for high-precision components, aiming at minimizing the fluctuation of assembly clearance and minimizing the remaining parts [11]. Xingyu et al. developed a new selective assembly method optimization for remanufactured machinery. Considering assembly accuracy, utilization of remanufactured parts, and assembly efficiency, Taguchi quality loss function (QLF) and cost function of remaining parts were both employed [12].

In terms of optimization algorithm, Asha et al. believed that each quality characteristic will fall in different groups in selective assembly, and used the non-dominated sorting genetic algorithm to find the best combination [13]. On this basis, selective group combinations for assembling the mating parts was obtained using particle swarm optimization (PSO) by Kannan [14]. However, the algorithm was demonstrated only for linear assembly, which consists of three components having equal dimensional distributions. Dong Z proposed a new selective assembly model used in multi-dimension chains and an improved PSO algorithm [15]. Liu et al. combined assembly accuracy and assembly rate to form a new optimization objective function, and studied the application of ant colony algorithm in computer aided selection assembly [16]. Ren Shuiping et al. proposed a selective assembly method based on Pareto and genetic algorithm to solve the problem of selecting mechanical products with multiple quality requirements [17]. 

The studies aforementioned merely focused on the assembly dimension chain and tolerance analysis, and take quality loss, assembly accuracy, and assembly rate as optimization objectives. However, the influence of non-uniform distribution of geometric error on mating surface is not addressed. In fact, due to the importance of assembly clearance when selecting shafts and holes, the uniformity of the assembly clearance needs to be taken as an optimization objective, which has not been fully considered in current researches. Therefore, this paper proposes an optimization method of selective assembly for shafts and holes based on relative entropy and dynamic programming. The relative entropy is used to evaluate the uniformity of assembly clearance, while dynamic programming is used to calculate the optimization results. Through this method, the optimal selective assembly plan can be obtained, which can provide guidance for the actual assembly. Besides, the influence of geometric error on assembly accuracy is reduced, and the final assembly accuracy and performance of products can be improved.

Our work is described in this paper, which is structured as follows. Related works are given in Section 2. Then, the method—where relative entropy has been used for evaluation and dynamic programming has been used as an optimization algorithm—is detailed in Section 3. A case is described in Section 4; results and discussion are shown in Section 5. Our conclusions and concepts for future work are presented in the final section.

## 3. Method

### 3.1. Selective Assembly Optimization Model for Shaft and Hole

**Figure 3:**
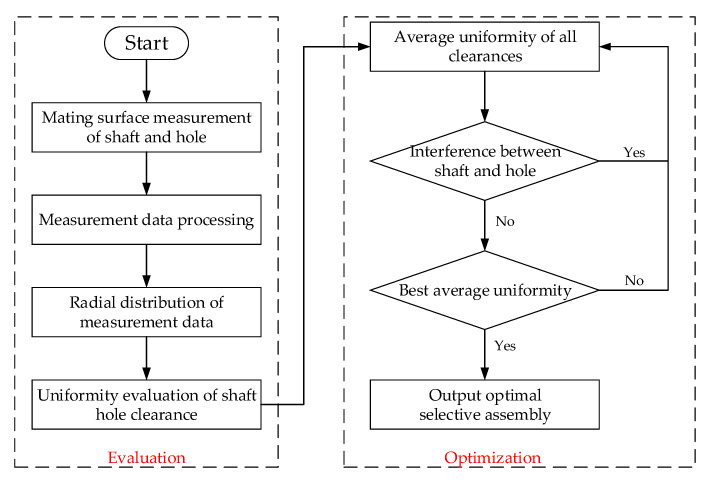
Selective assembly optimization model for shaft hole.

A selective assembly optimization model, intended to select the shaft and hole with uniform clearance, is proposed. In the model, the process has been broken down into two steps—evaluation and optimization—as illustrated in Figure 3.

In the first step, a series of measuring points characterizing geometric errors are measured using a coordinate measuring machine (CMM). The tactile CMM with high measurement accuracy (measurement uncertainty of 0.6 + L/600 μm) is selected in our work, according to the characteristics of small size and high machining accuracy for precision shaft and hole. The mating surface of shaft and hole is cylindrical, thus, on the premise of sampling theorem, several circles are measured along the axial direction, with dozens of points per circle.

After data processing, the radial distribution of geometric errors for shaft and hole is calculated. The radial distribution represents the ratio of the radius at a certain measurement point to the sum, which can be regarded as probability distribution. Relative entropy is an index to judge the difference between two probability distributions. The smaller its value, the smaller the difference, that is, the more uniform the clearance between shaft and hole. Therefore, the relative entropy is used to evaluate the assembly clearance uniformity of each pair of shaft and hole. 

In the second step, the dynamic programming has been used as an optimization algorithm. The average uniformity of all clearances is taken as the optimization objective, and the premise is that there is no interference between shaft and hole. Finally, the optimal pairing combinations are output for batch of shafts and holes.

### 3.2. Clearance Uniformity Evaluation of Shaft and Hole Based on Relative Entropy

Some studies have shown that entropy—which represents the average uncertainty of random events, and has been widely employed in control theory, image reconstruction, biology, and medicine, among others [18,19,20]—is more suitable for describing data uncertainty [21]. Preliminary research using information entropy to evaluate geometric errors on plane has been conducted [2,3,22,23]. The plane has been divided into a lattice with an *m* × *n* grid, *z_i,j_* represents the height of grid point (*P_ij_*), and the relative height at each point is *h_ij_* = *z_ij_ − z_min_*, where *z_min_* = min{*z_ij_*, *i* = 1, 2…, *m*; *j* = 1, 2, …, *n*}. The sum of the relative heights is therefore as shown in Equation (1):

(1) $$h=\sum_{i=1}^{m}\sum_{j=1}^{n}h_{ij}$$

The ratio ($h_{ij}^{\prime }$) of the height at a certain point to the sum is given as shown in Equation (2):

(2) $$\left\{ \begin{array}{l} h_{ij}^{\prime }=\frac{h_{ij}}{h}=\frac{h_{ij}}{\sum_{i=1}^{m}\sum_{j=1}^{n}h_{ij}}\\ \sum_{i=1}^{m}\sum_{j=1}^{n}h_{ij}^{\prime }=1,\;0\leq h_{ij}^{\prime }\leq 1 \end{array}\right.$$

If *h_ij_* is regarded as a sample of the random variable X, and $h_{ij}^{\prime }$ is regarded as sample probability, then information entropy can be used to reflect grid point height distribution. 

The following is a brief introduction to the principle of information entropy, which applies to the two subsequent levels of entropy evaluation.

Assume that a set of discrete random variables *X* = {*x*_1_, *x*_2_, …, *x_n_*}, and its probability distribution *p_i_* = *p*[*X* = *x_i_*] = {*p*_1_, *p*_2_, …, *p_n_*}; that is:

(3) $$\left[\begin{array}{c} X\\ p(x) \end{array}\right]=\left[\begin{array}{cccc} x_{1} & x_{2} & \cdots & x_{n}\\ p_{1} & p_{2} & \cdots & p_{n} \end{array}\right]\;0\leq p_{i}\leq 1,\sum_{i=1}^{n}p_{i}=1$$

Information entropy, *H*(*X*), characterizes the uncertainty of random events.

(4) $$H\left(X\right)=E\left(\log_{r}\frac{1}{p_{i}}\right)=\sum_{i=1}^{n}p_{i}\log_{r}\frac{1}{p_{i}}=-\sum_{i=1}^{n}p_{i}\log_{r}p_{i}$$

*E( )* indicates the mathematical expectation, and r is the base of the logarithm, which can take 2, e or 10. In our study, we use e as the base—allowing Equation (4) to be rewritten as shown in (5).

(5) $$H\left(X\right)=-\sum_{i=1}^{n}p_{i}\ln p_{i}$$

For the plane, the ratio of the height at a certain point to the sum is taken as the sample probability, and the information entropy is used to reflect the height distribution of measurement points. Similarly, for the cylindrical surface of shaft and hole, the ratio of the radius at a certain point to the sum can be taken as the sample probability, and the radial distribution of measurement points can be also reflected by entropy. Thus, Equations (1) and (2) can be rewritten as follows:

(6) $$R=\sum_{i=1}^{m}\sum_{j=1}^{n}R_{ij}$$

(7) $$\left\{ \begin{array}{l} R_{ij}^{\prime }=\frac{R_{ij}}{R}\\ \sum_{i=1}^{m}\sum_{j=1}^{n}R_{ij}^{\prime }=1,\;0\leq R_{ij}^{\prime }\leq 1 \end{array}\right.$$

where *m* and *n* represent the number of measurement points in the axial and radial directions, respectively. *r_i,j_* represents radius of measurement point, and the relative radius at each point is *R_ij_* = *r_i,j_ − r_min_*, where *r_min_* = min{ *r_i,j_*, *i* = 1, 2…, *m*; *j* = 1, 2, …, *n*}. $R_{ij}^{\prime }$ represents the ratio of the radius at a certain point to the sum.

Relative entropy is an index to judge the difference between two probability distributions on the basis of information entropy. The smaller its value, the smaller the difference, that is, the more uniform the clearance between shaft and hole.

If the probability distribution of shaft and hole are recorded as p(r) and q(r), respectively, then the relative entropy of the shaft and hole is given as shown in Equation (8):

(8) $$KL\left(p∥q\right)=\sum_{i=1}^{m}\sum_{j=1}^{n}p\left(r\right)\ln\frac{p\left(r\right)}{q\left(r\right)}$$

Notice, KL(p∥q)≠KL(q∥p).

### 3.3. Optimization Algorithm Based on Dynamic Programming

Relative entropy can be used to evaluate the assembly clearance of a pair of shaft and hole. However, there are several assembly options for batch of shafts and holes. Therefore, it is necessary to consider the comprehensive optimization objectives. Suppose a batch of shafts and holes are assembled; the number of shafts is *M*; the number of holes is *N*; there are *L* combinations of shafts and holes; $D_{i,j}^{k}$ indicates whether the *i*-th shaft and *j*-th hole are involved in the *k*-th combination, yes is 1, no is 0, where, *i* = 1,2,…, *M*; *j* = 1,2,…, *N*; *k* = 1,2,…, *L*. The constraint condition is that each part can be used for assembly at most once, and the quantity of each part must meet the assembly quantity. The mathematical description of the selective assembly optimization is as follows.

(9) $$\sum_{k=1}^{L}D_{i,j}^{k}\leq 1$$

(10) L≤min(M,N)

The optimization objective is to minimize the average relative entropy of all shafts and holes. Assuming that relative entropy of *k*-th combination is $f(D_{i,j}^{k})$, the optimization objective is shown in Equation (11):

(11) $$\min\sum_{k=1}^{L}f(D_{i,j}^{k})$$

Based on the above analysis, selective assembly of shaft and hole is essentially a kind of 0–1 programming problem, in which the variable either takes 0 or 1. Dynamic programming is suitable for solving such a problem. Through the optimization principle of dynamic programming, the multi-stage process is transformed into a series of single-stage problems. Based on the decision of the previous stage, the optimal scheme can be selected under the conditions of different stages, which can determine the optimal value [24]. Based on the idea of dynamic programming, there are many intelligent optimization algorithms, such as genetic algorithm, ant colony algorithm, simulated annealing algorithm, and so on. The cellular bat algorithm (CBA) [25] is used in our work, which can accelerate optimization speed and improve the ability of global optimization.

**Figure 4:**
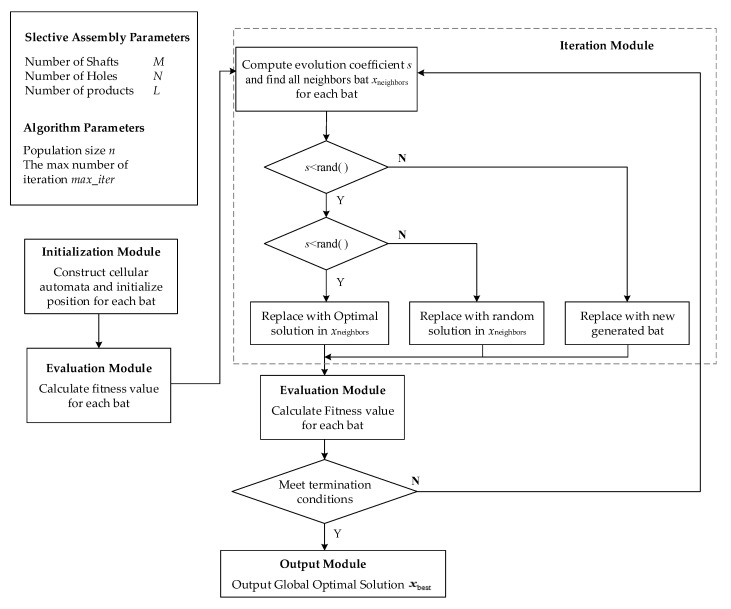
Cellular bat algorithm for selective assembly optimization.

The cellular bat algorithm consists of input module, initialization module, evaluation module, iteration module, and output module, as shown in Figure 4. 

The specific steps are as follows:

(1) Construct cellular automata and initialize parameters.

The number of shafts, holes, and products to be assembled are *M*, *N*, and *L*, where L≤min(M,N); The number of bat population is *n*; the position of the *i*-th bat is $x_{i}$, and its dimension is *L*. $x_{i}$ includes the assembly combination of *L* products. id_hole and id_axis, respectively, show the index of holes and shafts. The initialization method of $x_{i}$ is as follows:

(12) $$x_{i}^{j}\left(0\right)=\mathrm{id}\_\mathrm{hole}*M+\mathrm{id}\_\mathrm{axis}$$

where, j=1,2,…,L, id_hole∈{1,2,…,N},id_axis∈{1,2,…,M}. Equation (12) shows that in the zero iteration (the initialization stage), the *j*-th product is assembled by id_hole and id_shaft.

(2) Calculate the fitness of each bat in the initial population, and initialize the global optimal solution $x_{\mathrm{best}}$. The fitness represents the assembly evaluation of *L* products, which can be calculated by Equations (8) and (11).

(3) Update the position of each bat. 

① Calculate the evolution coefficient *s* and all neighbors $x_{\mathrm{neighbors}}$ of current bats by Equations (13) and (14).

(13) $$s=\frac{{\mathrm{fitness}}_{i}-{\mathrm{fitness}}_{\min}}{{\mathrm{fitness}}_{\max}-{\mathrm{fitness}}_{\min}}$$

(14) $$x_{\mathrm{neighbors}}=\left\{x|\mathrm{count}\_\mathrm{dif}\left(x,x_{i}\right)=1\right\}$$

where ${\mathrm{fitness}}_{i}$ is the fitness of the *i*-th bat; ${\mathrm{fitness}}_{\min}$ and ${\mathrm{fitness}}_{\max}$, respectively, show the min and max value of all fitness. In Equation (14), count_dif counts the number of different items in two equal-length vectors. This means that the neighbor bat and the current bat have only one item different from each other, and the other items are the same.

② If s<rand(), the current bat evolves into the optimal solution in $x_{\mathrm{neighbors}}$ with a certain probability, or is replaced by a solution in $x_{\mathrm{neighbors}}$ randomly. Where *s* and rand( ) range in [0, 1].

③ If s≥rand(), the current bat is replaced with new generated bat randomly.

(4) Recalculate the fitness of each bat and update the global optimal solution $x_{\mathrm{best}}$.

(5) Judge whether the termination condition is met. If so, output the global optimal solution; otherwise, turn to step (4). The termination condition is that the number of iterations is equal to the maximum number.

## 4. Case Study

**Figure 5:**
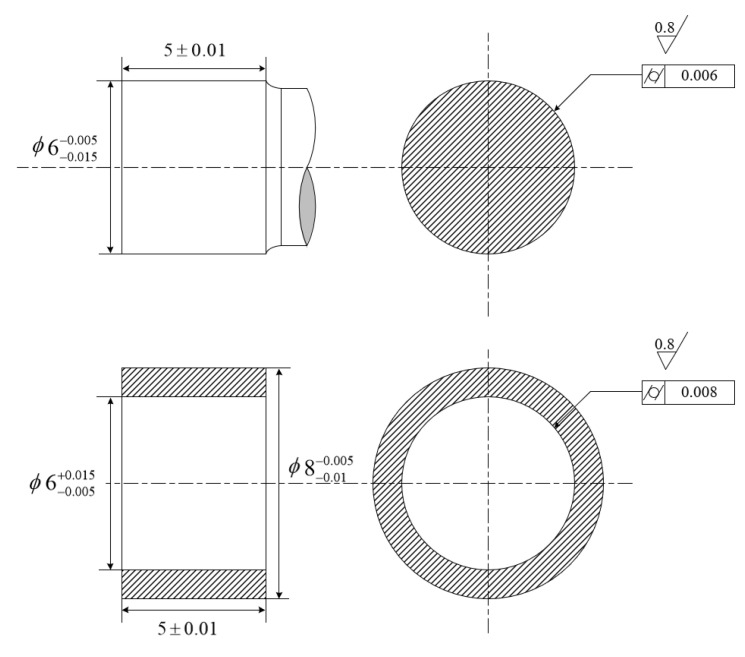
Explicit view of assembly components.

In this case, there are 8 shafts and 20 holes, which need to be assembled into 8 products. The explicit views of the parts-to-be-assembled with tolerances and dimensions are shown in Figure 5. The designed diameters of shafts and holes are 6 mm, and cylindricity error is 0.006 mm and 0.008 mm, respectively. The material of parts is beryllium alloy for aviation applications, which requires properties such as being lightweight, good stiffness, and stability. The material properties of beryllium alloy are listed as Table 1. In this case, the shaft-hole connection is under magnetic levitation. There is no direct contact between the assembly surfaces of the shaft and hole; and good coaxiality is required. Thus, no interference and clearance uniformity are regarded as optimization criterions. Their mating surfaces are measured using a tactile coordinate measuring machine (Leitz PMM866 of HEXAGON, measurement uncertainty of 0.6 + L/600 μm, measurement system of PC-DMIS). For each mating surface, 5 circles are measured along the axial direction, with 37 points per circle. The maximum and minimum radii of shafts and holes after the parts are numbered are listed in Table 2. 

The cylindricity of each part as per measurement data of shafts and holes is shown in Table 3. 

## 5. Results and Discussion

According to Equations (6) to (8), the relative entropy of each pair of shaft and hole is calculated; the results are shown in Table 4. (-) indicates that the maximum radius of the shaft is greater than the minimum radius of the hole, so it is not a clearance fit.

Using the optimization algorithm based on dynamic programming, the optimal combinations of selective assembly are listed in Table 5; average relative entropy is 0.1184. In the cellular bat algorithm, the larger the bat population, the greater the probability of obtaining the global optimal solution; the larger the maximum times of iteration, the longer the calculation time. Considering the optimization effect and time, this case uses the following parameters: the number of bat population is 100 and the maximum times of iteration is 200. 

For comparison, the traditional direct matching method based on tolerance is adopted. According to the cylindricity of holes shown in Table 3, eight holes with better cylindricity are selected and sorted from small to large, according to the minimum radius. At the same time, eight shafts are sorted from small to large, according to the maximum radius. Match shafts and holes by sorting, and check the interference of each pairing. Table 6 shows the matching results based on the direct matching method. The average relative entropy is 0.2330.

**Figure 6:**
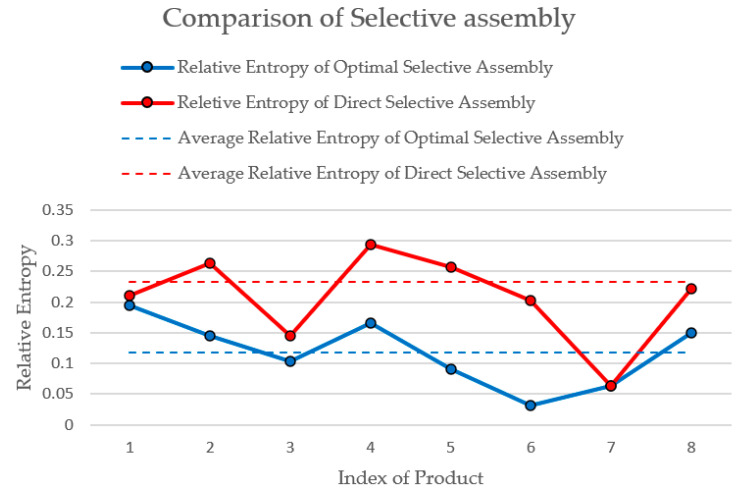
Comparison of two selective assembly results.

The comparison result of optimal selective assembly and traditional direct selective assembly is shown in Figure 6. The smaller the relative entropy, the more uniform the clearance between shaft and hole. The relative entropy of the seven products obtained by the two methods is same, but the average relative entropy of the eight products obtained by optimal selective assembly is smaller. Although direct selective assembly is effective to some extent, it cannot provide the best matching combination for eight products. The comparison shows that the global optimal result is obtained by the optimization method explained in this paper.

## 6. Conclusions and Future Work

This paper describes a selective assembly optimization method for shafts and holes based on relative entropy and dynamic programming. A selective assembly optimization model for shafts and holes was developed, including the evaluation and optimization process. Relative entropy is used to evaluate the assembly clearance uniformity of the shaft and hole. The average uniformity of all clearances is taken as the optimization objective, and dynamic programming has been used as an optimization algorithm. 

The proposed method is tested using 8 shafts and 20 holes. The proposed method was able to reflect the clearance uniformity between shafts and holes considering geometric errors, and could supplement traditional selective assembly.

In the future, the proposed method will be applied to the assembly of precision mechanical systems, such as inertial navigation systems and precision optical systems. The comprehensive optimization method of multi-batch assembly will be further considered, and the evaluation index of overall optimal assembly will be established to realize the optimal assembly accuracy of multi parts.

## Figures and Tables

**Table 1:** Material properties.

Material	Elastic Modulus (Gpa)	Poisson’s Ratio	Coefficient of Linear Expansion(/K^−1^)	Density (t/mm^3^)
Beryllium alloy	303	0.025	11 × 10^−6^	1.85 × 10^−9^

**Table 2:** Measurement results of shafts and holes (unit, mm).

Shafts			Holes					
No.	Minimum Radii	Maximum Radii	No.	Minimum Radii	Maximum radii	No.	Minimum Radii	Maximum Radii
1	2.994	2.997	1	3.0005	3.0031	11	2.9994	3.0022
2	2.9943	2.9966	2	2.9962	3.0047	12	2.9975	3.0031
3	2.9947	2.9961	3	3.0002	3.0082	13	3.0009	3.0029
4	2.9941	2.9964	4	3.0001	3.0034	14	3.0009	3.003
5	2.9922	2.9954	5	2.9991	3.0027	15	3.0009	3.0022
6	2.9936	2.9975	6	2.9982	3.0029	16	3.0004	3.0022
7	2.9939	2.9953	7	2.999	3.0036	17	2.9991	3.0014
8	2.9932	2.9971	8	2.9989	3.004	18	3.0008	3.0017
			9	3.0006	3.0036	19	2.9973	3.0008
			10	3.0015	3.0032	20	2.9998	3.0021

**Table 3:** Cylindricity of shafts and holes (unit, mm).

Shafts		Holes			
No.	Cylindricity	No.	Cylindricity	No.	Cylindricity
1	0.0035	1	0.0026	11	0.0069
2	0.0027	2	0.0071	12	0.0068
3	0.002	3	0.008	13	0.0036
4	0.0044	4	0.0033	14	0.005
5	0.0037	5	0.0036	15	0.0035
6	0.0027	6	0.0047	16	0.0035
7	0.0018	7	0.0046	17	0.0036
8	0.0044	8	0.0051	18	0.0019
		9	0.003	19	0.0027
		10	0.0047	20	0.0034

**Table 4:** Relative entropy of shafts and holes.

Holes\Shafts	1	2	3	4	5	6	7	8
1	0.2439	0.2255	0.1040	0.2245	0.1933	0.2368	0.0959	0.1919
2	-	-	-	-	-	-	-	-
3	0.4540	0.1721	0.1972	0.0912	0.1644	0.0318	0.1973	0.3017
4	0.4155	0.1445	0.2049	0.1722	0.2488	0.2054	0.2165	0.1576
5	0.2111	0.4210	0.2936	0.4207	0.3122	0.3057	0.3219	0.4468
6	0.2980	0.2615	0.1444	0.2580	0.2295	0.2583	0.1359	0.2382
7	0.1954	0.2641	0.1430	0.2727	0.2474	0.2643	0.1478	0.2312
8	0.3576	0.3352	0.1978	0.2938	0.1701	0.1904	0.2335	0.3884
9	0.3436	0.1877	0.2614	0.2629	0.3152	0.3056	0.2882	0.1497
10	0.2211	0.3112	0.2150	0.3507	0.3239	0.3500	0.2330	0.2552
11	0.4308	0.1642	0.2013	0.1791	0.2385	0.2023	0.2059	0.1807
12	0.5256	0.1674	0.2172	0.1663	0.2569	0.2026	0.2101	0.1974
13	0.2844	0.2621	0.1260	0.2533	0.1993	0.2478	0.1167	0.2357
14	0.4141	0.1807	0.2369	0.2038	0.2706	0.2162	0.2519	0.2067
15	0.2162	0.2773	0.1767	0.3022	0.2779	0.2997	0.1904	0.2370
16	0.1849	0.2554	0.1441	0.2202	0.0912	0.0948	0.1969	0.3254
17	0.4377	0.1970	0.2483	0.2162	0.2874	0.2273	0.2599	0.2226
18	0.3847	0.2124	0.2733	0.2752	0.3244	0.3062	0.2940	0.1877
19	0.2219	0.2063	0.0782	0.1989	0.1721	-	0.0641	0.1682
20	0.2344	0.2825	0.1683	0.3069	0.2845	0.3191	0.1716	0.2294

**Table 5:** Optimal selective assembly of shafts and holes.

Index of Shafts	Index of Holes	Relative Entropy
1	7	0.1954
2	4	0.1445
3	1	0.1040
4	12	0.1663
5	16	0.0912
6	3	0.0318
7	19	0.0641
8	9	0.1497
Average relative entropy		0.1184

**Table 6:** Direct selective assembly of shafts and holes.

Index of Shafts	Index of Holes	Relative Entropy
7	19	0.0641
5	12	0.2569
3	6	0.1444
4	8	0.2938
2	7	0.2641
1	5	0.2111
8	17	0.2226
6	11	0.2023
Average relative entropy		0.2074
